# Melanoacanthoma Masquerading as Melanoma: Case Reports and Literature Review

**DOI:** 10.7759/cureus.4998

**Published:** 2019-06-25

**Authors:** Nikolas Gutierrez, Christof P Erickson, Antoanella Calame, Brooke R Sateesh, Philip R Cohen

**Affiliations:** 1 Family Medicine, Touro University College of Osteopathic Medicine, Vallejo, USA; 2 Dermatology, Compass Dermatopathology, Inc., San Diego, USA; 3 Dermatology, San Diego Family Dermatology, National City, USA

**Keywords:** melanoacanthoma, melanoma, malignant, pigmented melanocyte

## Abstract

Melanoacanthoma is a benign epithelial tumor composed of melanocytes and keratinocytes that can morphologically mimic malignant neoplasms. Two patients with melanoacanthoma that clinically masqueraded as melanoma are described: a 65-year-old African-American woman with a pigmented nodule on the right preauricular area and an 85-year-old Haitian-Creole man with a large exophytic nodule on his left lower abdomen. Melanoma was clinically suspected in both patients. Biopsies were performed, which established the diagnosis of melanoacanthoma. Complete removal of a melanoacanthoma should be considered since partial excision may result in recurrence.

## Introduction

Melanoacanthoma is a benign, cutaneous neoplasm consisting of melanocytes and keratinocytes [1]. It is a solitary lesion that may occur on the head, neck, trunk, and extremities; in addition, it does not have a predilection for either race or sex [2-4]. Melanoacanthoma can morphologically mimic the appearance of melanoma, creating a diagnostic challenge for clinicians [1]. Histological evaluation of melanoacanthoma reveals hyperkeratosis, acanthosis, and papillomatosis with large, hyperpigmented, dendritic melanocytes spanning the epidermis [1,3,5]. Two patients whose melanoacanthoma masqueraded as melanoma are described and the salient features of melanoacanthoma are reviewed.

## Case presentation

Case 1 

A 65-year-old African-American woman with a history of lymphoma, currently in remission, presented with a black lesion on the right side of her face. The lesion had been progressively enlarging and had begun to irritate her skin. Her past medical history was remarkable for arthritis, asthma, depression, hypertension, and insomnia. She also had a history of hand dermatitis but denied any other personal or family history of skin cancer. She smoked one pack of cigarettes per day and did not drink alcohol. 

**Figure 1:**
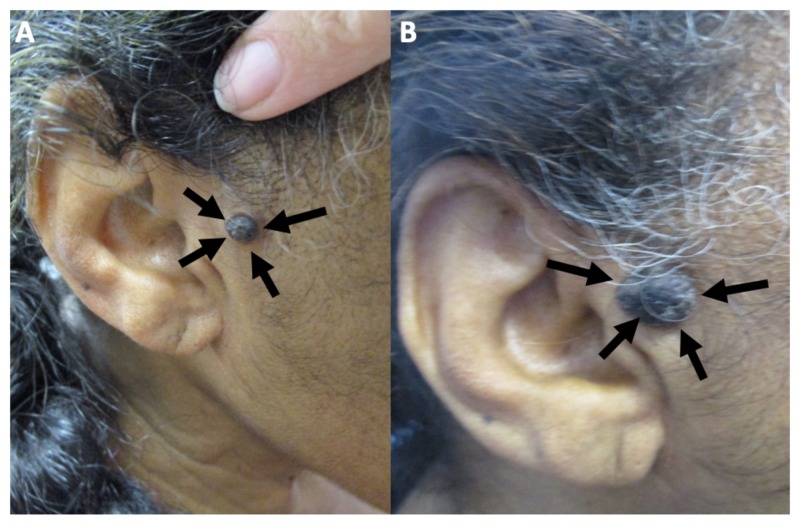
Clinical presentation of melanoacanthoma on the right preauricular area of a 65-year-old woman Clinical presentation of the initial (A) and recurrent (B) melanoacanthoma (black arrows) on the right preauricular area. The tumor initially presented as a 2 x 1-cm black plaque (A). The lesion not only persisted but also increased in size, morphologically mimicking a melanoma (B).

A complete cutaneous examination was performed. A 2 x 1-cm black plaque was present in the right preauricular area (Figure 1). A superficial shave biopsy of the upper portion of the lesion was performed. An intradermal nevus was considered in the differential diagnosis. 

**Figure 2:**
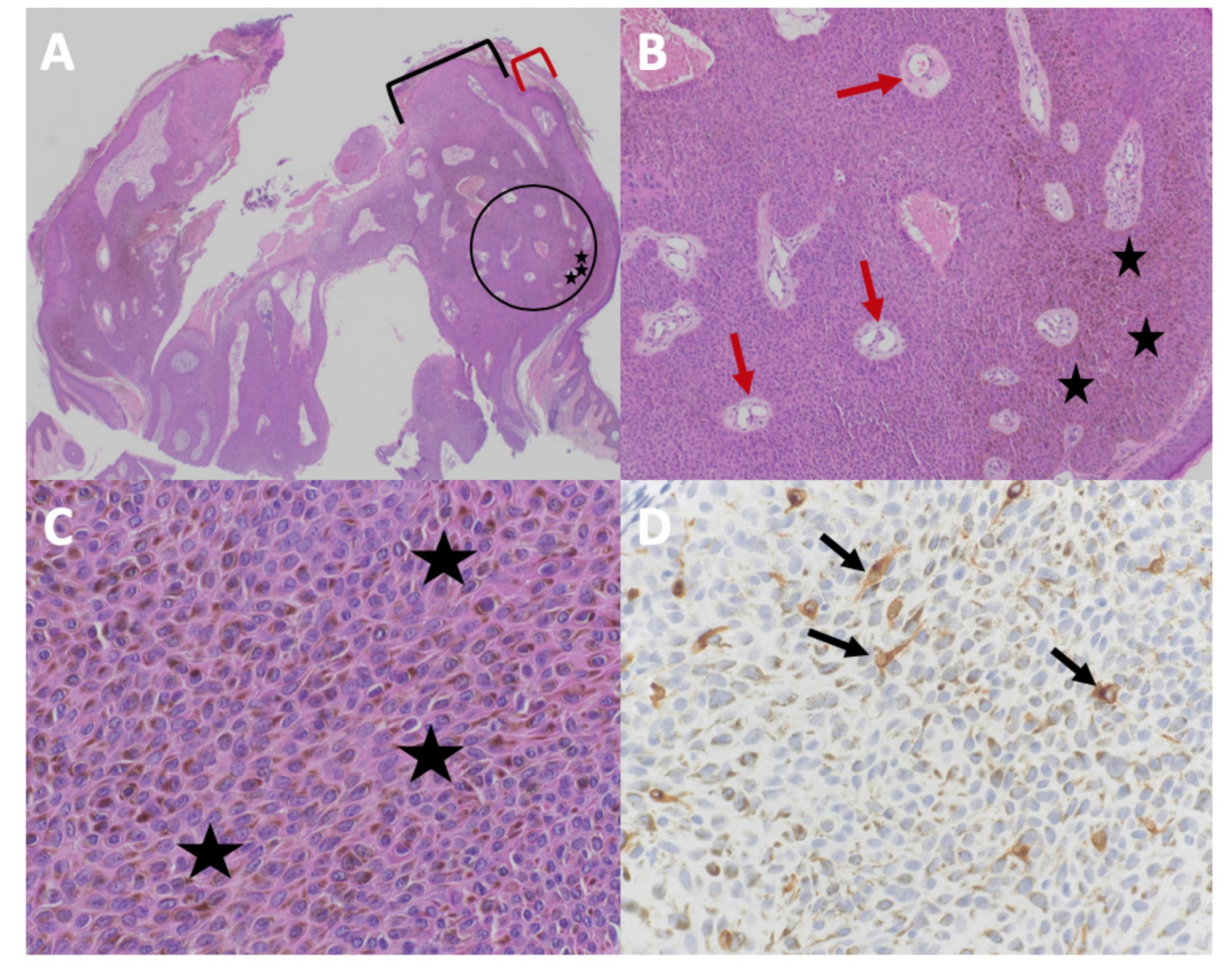
Microscopic presentation of melanoacanthoma on the right preauricular area of a 65-year-old woman Distant (A) and closer (B-D) views of pathologic features of a melanoacanthoma. Low (A) and higher (B and C) magnification of hematoxylin and eosin stained sections shows an exophytic nodule with hyperkeratosis (thickening of the stratum corneum as shown between the red bracket) and acanthosis (thickening of the epidermis as shown between the black bracket); the area enclosed in the black circle of image A is shown at higher magnification in image B. There is hyperpigmentation throughout all layers of the epidermis (black stars). Tangential sectioning of the tumor shows small areas of dermis, containing epithelial lined vessels and erythrocytes within the epithelium (red arrows). There is lymphocytic perivascular inflammation in the dermis. A higher magnification view (D) of MART-1 stained section shows positive staining of dendritic melanocytes throughout all layers of the epidermis (black arrows) (Hematoxylin and eosin: A, x2; B, x10; C, x40; MART-1 immunoperoxidase; D, x40).

Microscopic evaluation of the biopsy specimen revealed acanthosis with a uniform proliferation of heavily pigmented basaloid cells extending into the upper epidermis. There was variable maturation of the epithelium and occasional mitoses. Heavily pigmented melanophages and inflammation were present in the dermis (Figure 2). Immunohistochemical stains revealed dendritic melanocytes with MART-1. 

Correlation of the clinical morphology and pathologic findings established the diagnosis of an irritated melanoacanthoma. Given the benign nature and good prognosis of melanoacanthoma, the patient denied further intervention.

**Figure 3:**
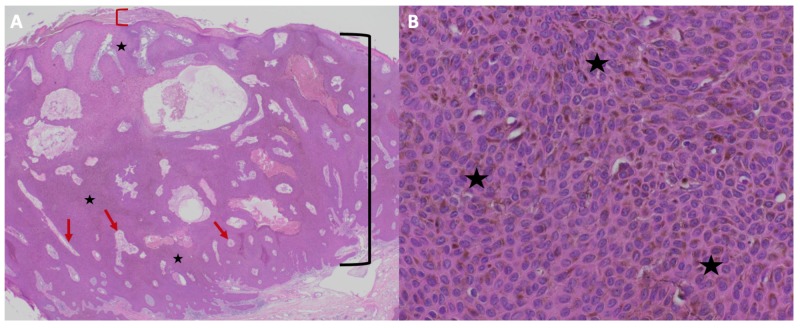
Microscopic presentation of recurrent melanoacanthoma on the right preauricular area of a 65-year-old woman Distant (A) and closer (B) views of pathologic features of a melanoacanthoma. Low (A) and higher (B) magnification of hematoxylin and eosin stained sections show an exophytic nodule with hyperkeratosis (thickening of the stratum corneum as shown between the red bracket) and acanthosis (thickening of the epidermis as shown between the black bracket). There is hyperpigmentation throughout all layers of the epidermis (black stars). Tangential sectioning of the tumor shows small areas of dermis, containing epithelial lined vessels and erythrocytes within the epithelium (red arrows). There is lymphocytic perivascular inflammation in the dermis (Hematoxylin and eosin: A, x2; B, x40).

The patient returned several months later; the lesion persisted and continued to increase in size (Figure 1). In order to confirm the diagnosis and exclude the possibility of malignant melanoma, a deep shave biopsy was performed. The pathological findings on both hematoxylin and eosin stain and immunoperoxidase stained sections were the same as those observed on her initial biopsy (Figure 3). Correlation of clinical and pathological findings reaffirmed the diagnosis of melanoacanthoma. The biopsy site healed without recurrence of the lesion.

Case 2

An 85-year-old Haitian-Creole man presented with an asymptomatic mass on his left hip. The lesion had been present for over a year and has been progressively enlarging. He had a history of hepatitis B and hepatitis C but denied personal or family history of melanoma or other skin cancers. He was retired and had no history of tobacco or alcohol abuse. A comprehensive systemic review revealed no pertinent abnormalities. 

**Figure 4:**
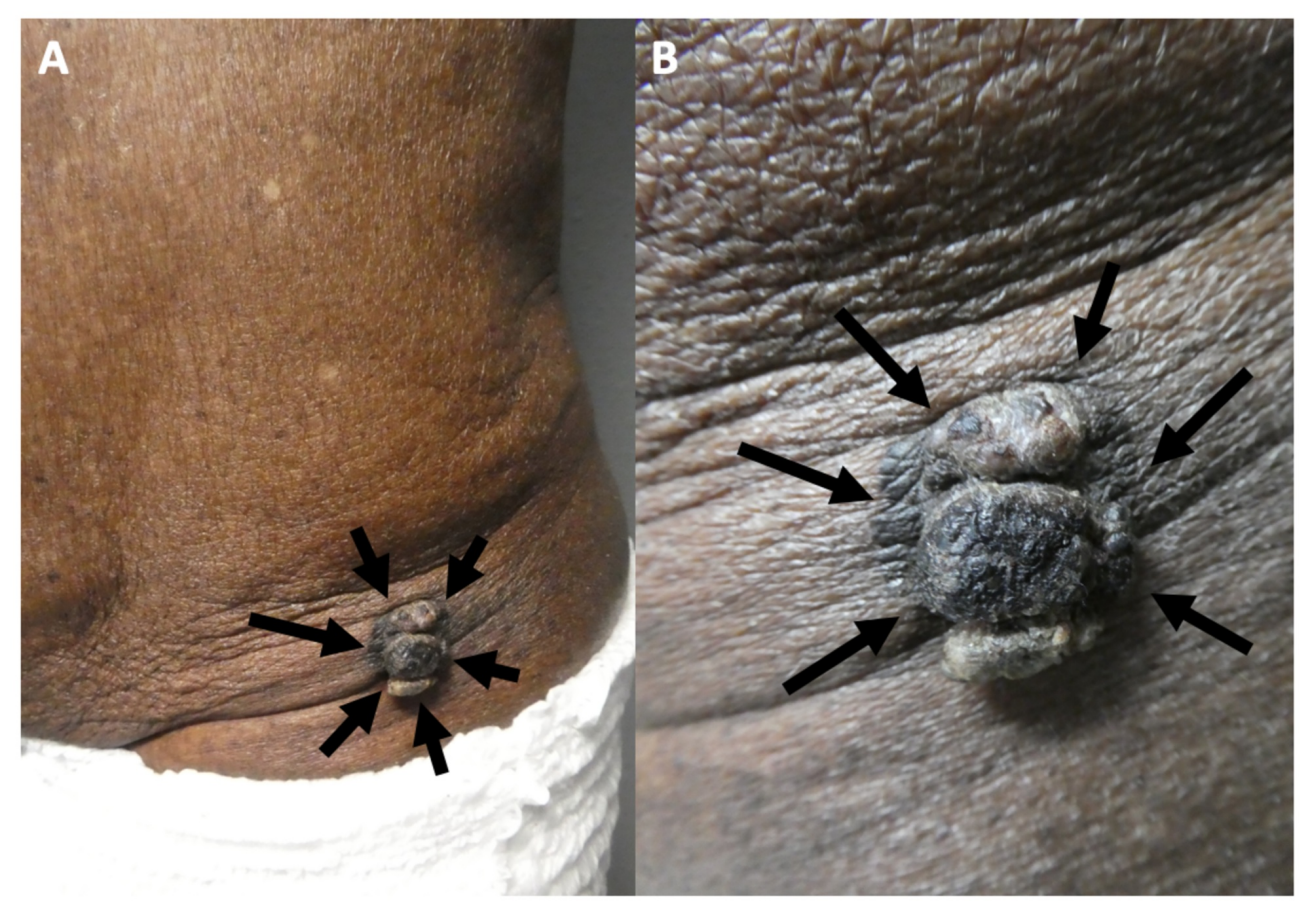
Melanoacanthoma on the left lower abdomen of an 85-year-old man Distant (A) and closer (B) views of a melanoacanthoma (black arrows) presenting as a large 3 x 2.5-cm lobulated, exophytic, black nodule with hyperpigmentation of the surrounding skin on the left lower abdomen.

A complete cutaneous examination was performed and revealed a large 3 x 2.5-cm lobulated, exophytic, black nodule with hyperpigmentation of the surrounding skin on his left lower abdomen (Figure 4). The initial clinical impression favored a nodular malignant melanoma and an excisional shave biopsy was performed. There was no palpable neck, axilla, or inguinal lymphadenopathy. 

**Figure 5:**
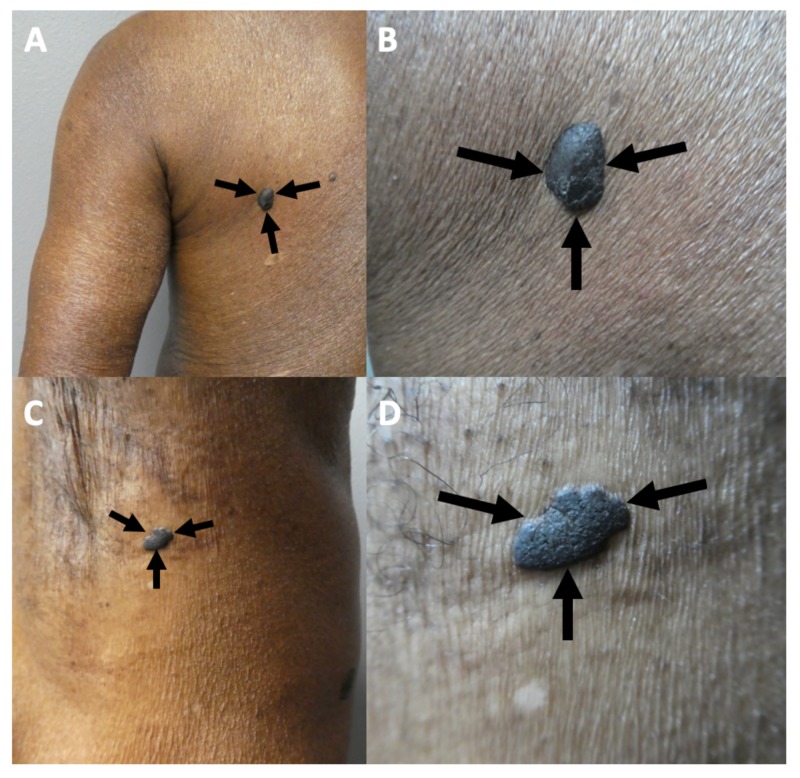
Pigmented seborrheic keratoses on the left posterior shoulder and the left axilla on an 85-year-old man. The left posterior shoulder (A and B) and left axilla (C and D) show distant (A and C) and closer (B and D) views of pigments seborrheic keratoses (black arrows) in a man with concurrent melanoacanthoma on his left lower abdomen.

Black plaques were present on his left posterior shoulder measuring 15 x 10 mm and his left axilla measuring 10 x 5 mm (Figure 5). The initial clinical impression of these lesions was pigment seborrheic keratoses. Excisional shave biopsies of both lesions were done. 

**Figure 6:**
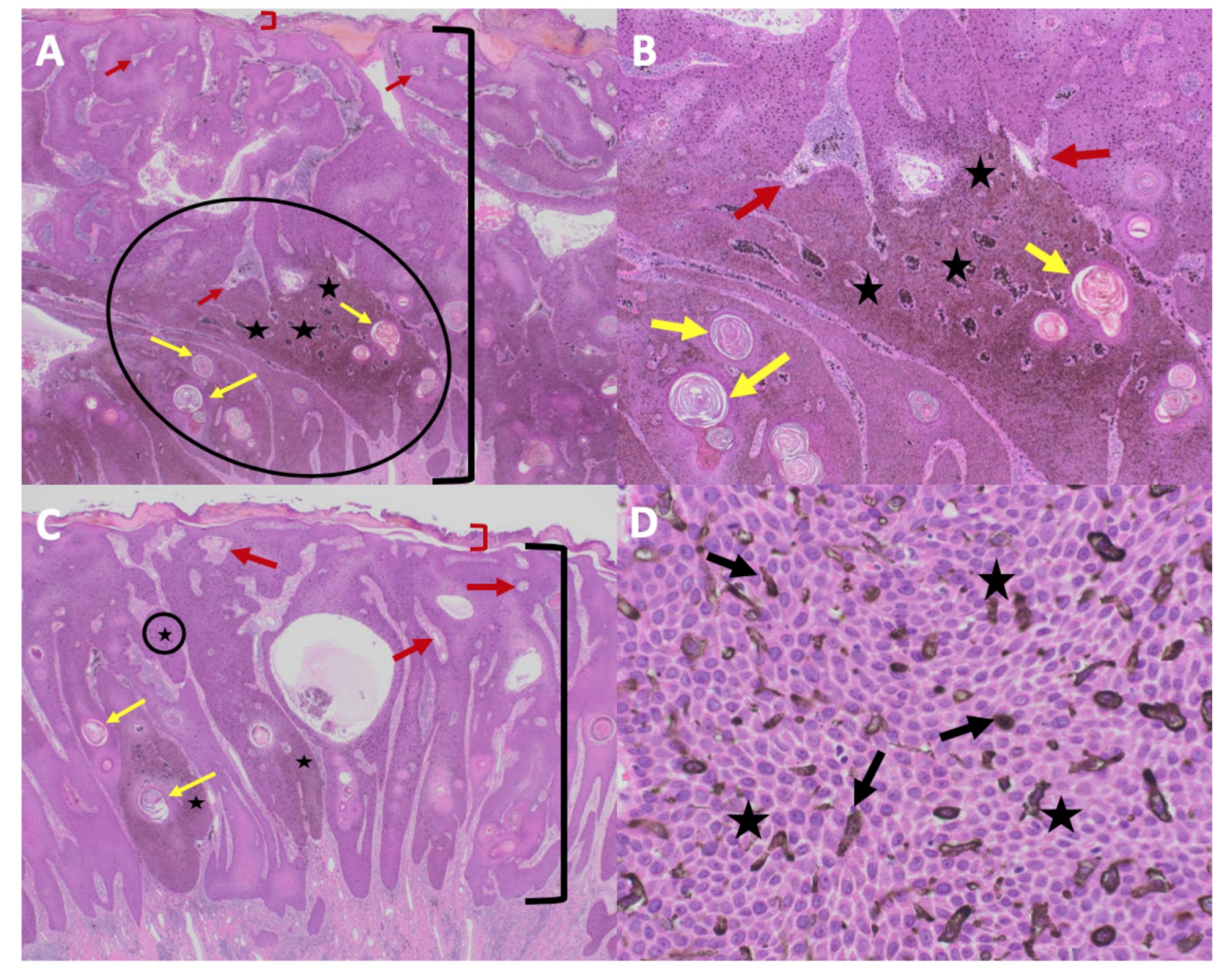
Microscopic presentation of melanoacanthoma on the left lower abdomen of an 85-year-old man The melanoacanthoma on the lower abdomen of an 85-year-old man contains exophytic (A and B) and endophytic (C and D) portions. Low (A) and higher (B) magnification of the exophytic portion shows hyperkeratosis (thickening of the stratum corneum as shown between the red bracket) and acanthosis (thickening of the epidermis as shown between the black bracket); the area enclosed in the black circle of image A is shown at higher magnification in image B. There is hyperpigmentation throughout all layers of the epidermis (black stars). Tangential sectioning of the tumor shows not only keratin-filled pseudocysts (yellow arrows) but also small areas of dermis containing epithelial lined vessels and erythrocytes within the epithelium (red arrows). There is lymphocytic perivascular inflammation in the dermis. Low (C) and higher (D) magnification of the endophytic portion shows similar pathologic changes; the area enclosed in the black circle of image C is shown at higher magnification in image D. In addition, the higher magnification view (D) shows dendritic melanocytes throughout all layers of the epidermis (black arrows) and hyperpigmentation throughout all layers of the epidermis (black stars; Hematoxylin and eosin: A, x2; B, x10; C, x40; D, x40).

Microscopic evaluation of the left lower abdomen tumor revealed papillomatosis, acanthosis, and heavily pigmented melanocytes throughout the epidermis (Figure 6). There was also heavily pigmented melanophages in the dermis. Correlation of the clinical morphology and pathologic findings of the left lower abdomen nodule established the diagnosis of melanoacanthoma. 

**Figure 7:**
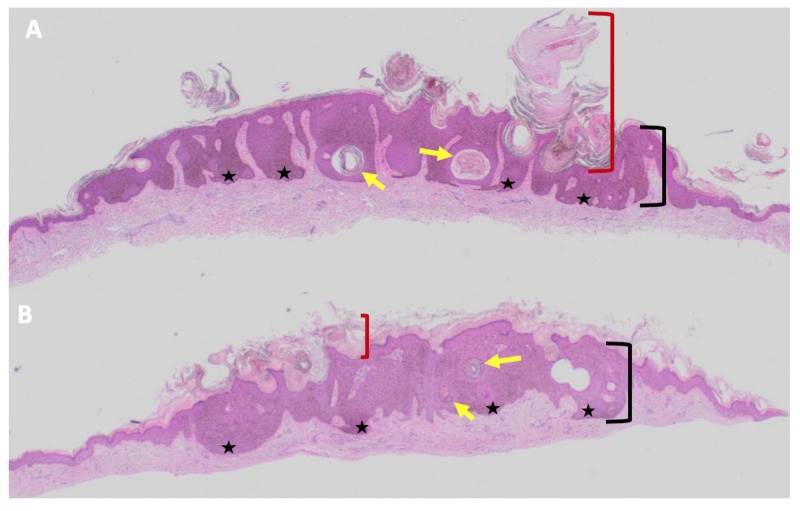
Microscopic presentation of pigmented seborrheic keratoses on the left posterior shoulder and left axilla of an 85-year-old man with concurrent melanoacanthoma on his left lower abdomen. The left posterior shoulder (A) and left axilla (B) show complete views of pigmented seborrheic keratoses in an 85-year-old man. Pathologic features include hyperkeratosis (thickening of the stratum corneum as shown between the red bracket), acanthosis (thickening of the epidermis as shown between the black bracket), and hyperpigmentation restricted to the basal layers of the epidermis (black stars). Tangential sectioning of the tumor also shows only keratin-filled pseudocysts (yellow arrows) (Hematoxylin and eosin: A, x2; B, x2).

Microscopic examination of the left posterior shoulder and left axilla both revealed acanthosis with heavy melanin deposits in the dermis (Figure 7). The entire lesions were removed with the biopsies. The clinical morphology and pathologic features established a diagnosis of pigmented seborrheic keratosis. 

All of the biopsy sites healed without complication and follow-up examination did not reveal recurrence of the lesions. 

## Discussion

Melanoacanthoma is a rare, benign neoplasm consisting of melanocytes and keratinocytes. This lesion was first described by Bloch as a non-nevoid melanoepithelioma; he also further categorized the tumor into type 1 and type 2 lesions. Mishima and Pinkus subsequently identified two different types of neoplasms. They coined the term melanoacanthoma to describe the type 1 lesion and identified the type 2 lesion as a pigmented seborrheic keratosis [2]. 

Melanoacanthoma is rare. It was estimated that this lesion occurs in only five of 500,000 people [6]. Melanoacanthoma tends to occur in elderly individuals, with a median age of 65 years and shows no sex predilection [1,4-7]. 

Melanoacanthoma was originally noted to occur more commonly in light-skinned individuals [5-7]. Although less frequent, similar to our patients, melanoacanthoma has also been reported in dark-skinned individuals [4,5,7]. Hence, whether there is a race predilection for this tumor remains to be determined.

The presentation of melanoacanthoma is variable. Typically, it is a painless, slow growing, solitary lesions with an increased proclivity to occur in areas of trauma. It ranges from flat to raised, light-brown to black and has a stuck-on appearance. 

Classically, cutaneous melanoacanthoma is pigmented and presents as either a papule, plaque, nodule or horn [5,7-8]. It typically occurs on the head, neck, trunk, and extremities. However, it has also been observed on the penile shaft and the genital region [5,9]. 

Melanoacanthoma greater than 2.5 centimeters in diameter has been described as large [6] while giant lesions have been reported that measure ten to 12 centimeters [4,7]. The rate at which the lesion enlarges widely varied. However, many of the patients with large and giant lesions had their tumor for several years before they had sought medical attention [4,6-8]. 

Establishing the diagnosis of a melanoacanthoma based only on the morphologic features of the lesion may be difficult. Dermoscopy may be a useful tool. The dermoscopic features of melanoacanthoma include a sunburst pattern and ridges and fissures [4,10-11].

In a review of eight patients with melanoacanthomas, Chung et al. found that all of the lesions had dermoscopic features of seborrheic keratoses (comedo-like openings, sharp demarcations, milia-like cysts, moth-eaten border, and hairpin vessels). However, six lesions also had melanoma-specific dermoscopic features (blue-white veil, atypical dots, granularity, and polymorphous vessels) [1]. Therefore, since melanoacanthoma can have similar dermoscopic findings to those observed in melanoma, a biopsy may be necessary to establish the diagnosis [10]. 

Melanoacanthoma can mimic other skin lesions. The clinical differential diagnoses of melanoacanthoma are listed in Table 1 [1,3,5,11-13]. However, melanoacanthoma is readily identified on histologic evaluation. 

**Table 1:** Clinical differential diagnoses of melanoacanthoma

Condition	Description	References
Actinic keratosis	Solitary ill-defined, scaly, rough, red plaque	[1]
Condyloma accuminata	Smooth-surfaced exophytic papilloma that may be skin colored, brown, or white; it typically occurs in the genital region	[5]
Lentigo maligna	Melanoma in situ with lentiginous features occurring in elderly individuals on sun-damaged skin with potential to transform to melanoma	[3]
Melanocytic nevus	Black or brown macule or papule with well-defined borders; it can be congenital or acquired	[13]
Melanoma	Pigmented lesion with asymmetry, poorly demarcated borders, variable coloration, and diameter that has increased in size	[1]
Pigmented spindle cell (Spitz) nevus	Dark black or brown macule or papule; it is often less than 6 millimeters in diameter	[11]
Seborrheic keratosis	Well-defined papule or plaque with a waxy hyperkeratotic surface; it may be flesh-colored or pigmented	[1]
Solar lentigo	Brown macule or patch with sharp margins on sun-exposed skin	[1]
Verrucous carcinoma	Warty-like plaques or nodules which may appear on the distal extremities (plantar feet or finger) or groin area; it is a rare variant of squamous cell carcinoma	[12]
Verruca vulgaris	Hyperkeratotic, filiform papules or plaques that may have black spots from thrombosed capillaries	[12]

Hematoxylin and eosin staining of melanoacanthoma shows papillomatosis, acanthosis, and hyperkeratosis with heavily pigmented melanocytes dispersed throughout all layers of the epidermis. Other features commonly seen include keratin-filled pseudocysts, inflammation, and Langerhans cells [3,5]. Electron microscopy confirms the presence of dendritic melanocytes [3]. 

Dopa and Fontana-Masson silver stains can be used to differentiate melanoacanthoma from seborrheic keratosis. Both of these stains highlight melanin and melanocytes in all layers of the epithelium in melanoacanthoma. In contrast, positive staining is confined to the basal layers of the epidermis in seborrheic keratosis [3]. 

Immunoperoxidase staining may be useful in establishing the diagnosis of a melanoacanthoma. Indeed, using a chromogen with a red indicator may also be helpful in identifying melanoacanthoma by differentiating melanocytes from melanin-laden keratinocytes. Cytokeratin such as CK7, which shows positive staining of the keratinocytes in melanoacanthoma, is a marker that can aid in differentiating melanoacanthoma from melanoma [14]. 

Melanoma antigen recognized by T-cells 1 (MART-1) is an antigen that highlights melanocytes. It demonstrates positive staining of the melanocytes in a melanoacanthoma. However, since melanoma and benign nevi also demonstrate positive staining of melanocytes with MART-1, other pathologic features observed on hematoxylin and eosin staining are necessary to confirm the diagnosis of melanoacanthoma [13,15].

Many investigators consider melanoacanthoma to be a variant of seborrheic keratosis. Yet, the definitive pathogenesis of melanoacanthoma remains to be established. Electron microscopy studies suggest that the presence of highly dendritic, hyperpigmented melanocytes is due to a failure of melanin transfer from melanocytes to keratinocytes. Variation in the arrangement and speed of keratinocyte differentiation has been speculated as the etiology of this failed transfer of melanin. In addition, Langerhans cells, which are present throughout the Malpighian layers of the epidermis, have been found to affect the proliferation control of keratinocytes and may play a role in the failed transfer [3,16]. 

The proclivity of melanoacanthoma to occur in areas of prior trauma introduces the possibility of cutaneous injury as a factor in their pathogenesis. One of our patients not only had a melanoacanthoma but also two seborrheic keratoses. The observation of concurrent pigmented seborrheic keratoses and melanoacanthoma prompts speculation that the pathogenesis may be different than that of seborrheic keratosis.

Excisional biopsies of melanoacanthomas can be both diagnostic and curative. Although these benign neoplasms do not need to be removed, most patients opt for their lesion to be excised for cosmetic purposes or because their skin is irritated by the tumor -- similar to our female patient. Incomplete removal of the lesion at the time of biopsy may result in recurrence and require subsequent intervention. Patients with melanoacanthomas have a good prognosis with low risk of recurrence following complete removal of the tumor. 

## Conclusions

Melanoacanthoma is a benign tumor consisting of melanocytes and keratinocytes that typically occurs in older individuals and presents as a light-brown to black plaques or nodules ranging in size from two by two millimeters to 15 by 15 centimeters. Similar to our patients, the clinical differential diagnosis of melanoacanthoma includes not only melanoma, but also other melanocytic neoplasms and epithelial tumors; microscopic examination establishes the diagnosis. The concurrent observation of pigmented seborrheic keratosis and melanoacanthoma in one of our patients prompts the speculation that the pathogenesis of melanoacanthoma may be different than that of a seborrheic keratosis. Complete, conservative excision of the tumor is the treatment of choice; partial removal may result in recurrence as observed in one of our patients.
